# Bidirectional chemotherapy combining intraperitoneal docetaxel with intravenous 5-fluorouracil and oxaliplatin for patients with unresectable peritoneal metastasis from gastric cancer: the first study in Western countries

**DOI:** 10.1515/pp-2019-0035

**Published:** 2020-04-17

**Authors:** Rea Lo Dico, Jean Marc Gornet, Nicola Guglielmo, Aziz Zaanan, Julien Taieb, Marc Pocard

**Affiliations:** University of Paris, UMR 1275 CAP Paris-Tech, Department of Digestive Surgery, Lariboisière Hospital, AP-HP, F-75010 Paris, France; Department of Hepatology, Gastroenterology and Digestive Oncology, Saint-Louis Hospital, AP-HP, Paris, France; Department of Digestive Surgery, Lariboisiere Hospital, AP-HP, Paris, France; Department of Hepatology, Gastroenterology and Digestive Oncology, Georges Pompiodou European Hospital, AP-HP, Paris, University of Paris, France; University of Paris, UMR 1275 CAP Paris-Tech, Department of Digestive Surgery, Lariboisière Hospital, AP-HP, F-75010 Paris, France

**Keywords:** advanced gastric cancer, bidirectional chemotherapy, cytoreductive surgery, HIPEC, intraperitoneal chemotherapy, peritoneal metastasis

## Abstract

**Background:**

A new treatment using bidirectional intraperitoneal (IP) and intravenous (IV) chemotherapy developed by Asiatic surgeons improves outcomes in patients with synchronous peritoneal metastasis (PM) from gastric cancer (GC).

**Methods:**

We enrolled six consecutive patients with unresectable PM from GC who underwent bidirectional chemotherapy using IP docetaxel and IV FOLFOX or LV5FU2. In one course, IP docetaxel 30 mg/m^2^ was administrated on days 1, 8 and 15, and IV FOLFOX or LV5FU2 was administered on days 1 and 15, followed by 7 days of rest. Before and after a complete bidirectional cycle of three courses, the peritoneal cancer index (PCI) was evaluated by laparoscopy. The primary endpoint was to evaluate the feasibility and safety of bidirectional chemotherapy. Secondary endpoints were overall survival (OS), and the success of the therapeutic strategy was reflected by a decrease of 25% of the initial PCI.

**Results:**

All patients completed one bidirectional cycle. The regimen was well tolerated. The median OS was 13 months [range 5–18], and the 1-year OS rate was 67%. After the first bidirectional cycle, the PCI decrease ≥25% of the initial value in four patients. A major histological response was observed in four patients.

**Conclusions:**

This is the first Western study and confirms the feasibility and safety of bidirectional treatment using IP and IV chemotherapy for patients with unresectable PM from GC, resulting in a 13-month median OS with limited morbidity. The decrease in PCI after one bidirectional cycle is promising.

## Introduction

More than 50% of patients with advanced gastric cancer (GC) die of peritoneal recurrences. Peritoneal metastasis (PM) is frequent (in up to 20% of patients), induces symptoms and often limits treatment options. The median overall survival (OS) of patients with PM from GC treated with systemic chemotherapeutic agents such as taxanes, platinum salts and 5-fluorouracil (5-FU) is poor, between 3 and 8 months for HER-2 negative tumours [1, 2, 3]. Despite recent advances [4, 5], this limited survival has not truly increased in recent years, and new treatment options are required.

Several reports have suggested that peritonectomy and cytoreductive surgery (CRS) combined with hyperthermic intraperitoneal chemotherapy (HIPEC) and/or postoperative intraperitoneal chemotherapy may cure selected patients with PM from various digestive and extra-digestive cancers [6, 7]. However, for PM of gastric origin, the efficiency of this combined procedure remains highly controversial. The experience of a few institutions has yielded encouraging survival results in patients treated with CRS combined with HIPEC [8, 9]. Moreover, many patients are not candidates for such treatment and are consequently treated with palliative systemic chemotherapy only.

In such patients, Asiatic surgeons have recently proposed a new treatment using neoadjuvant intraperitoneal and systemic chemotherapy that is associated with a high response rate and low toxicity [10, 11, 12, 13, 14, 15, 16, 17, 18]. This bidirectional treatment combines intraperitoneal (IP) administration of docetaxel and intravenous (IV) administration of 5-FU or oral administration of S-1. Japanese authors claimed that such chemotherapeutic agent combinations, known to be effective for GC, could increase the rate of patients eligible for CRS and HIPEC procedures and potentially offer curative approaches with acceptable toxicity [19]. However, GC in Western countries is considered differently from GC in Japan in terms of its epidemiology and possibly its biology and clinical response to surgery [20]. Moreover, oral S-1 administration was considered inefficient in European Caucasian patients and is not used in Europe. We planned a novel therapeutic strategy for uses in Western countries combining IP administration of docetaxel and IV administration of FOLFOX (LV5FU2 with oxaliplatin) in patients with unresectable PM from GC to facilitate the setup of a phase I trial. The main endpoint of our study was to evaluate the feasibility and safety of this neoadjuvant bidirectional treatment. The secondary endpoint was to evaluate the OS and the success of the therapeutic strategy as reflected by a decrease of 25% of the peritoneal spread as evaluated by laparoscopy.

## Materials and methods

### Patients

**Figure 1:**
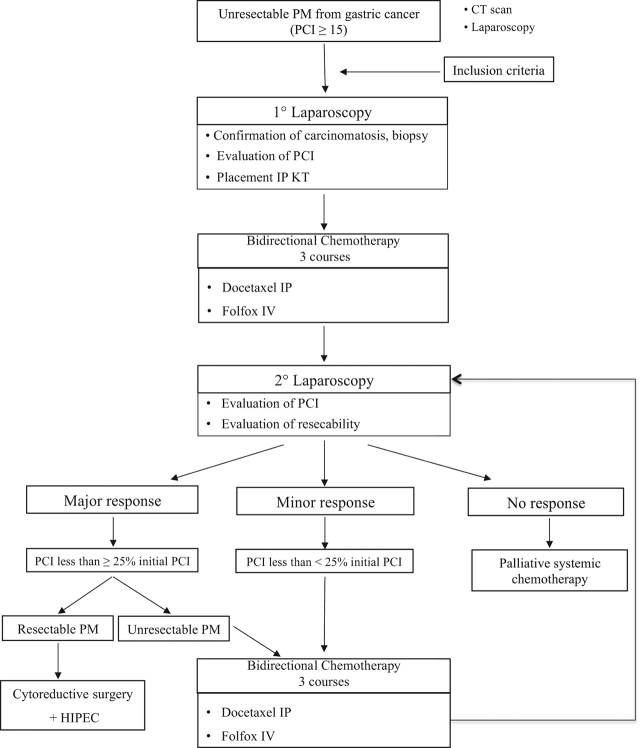
Trial profile.

PM, peritoneal metastasis; PCI, peritoneal cancer index; CT scan, computed tomography scan; KT, catheter; IV, intravenous; IP, intraperitoneal; HIPEC, hyperthermic intraperitoneal chemotherapy; FOLFOX, folinic acid, fluorouracil and oxaliplatin.

All consecutive patients with PM from GC were included in this first study in Western countries. This prospective feasibility study was performed to test intraperitoneal docetaxel. The Oncological Review Board and Ethics Committee approved the indication of the strategy in accordance with the ethical standards of the Helsinki Declaration of 2013. Informed consent according to the Institutional Guideline was obtained for all patients prior to the trial. The inclusion criteria of the patients were as follows: extended synchronous or metachronous PM from gastric adenocarcinoma considered unresectable with a peritoneal cancer index (PCI) ≥ 15; histologically proven gastric adenocarcinoma; absence of haematogenous metastases and remote lymph node metastases; Eastern Cooperative Oncology Group (ECOG) status of 0 to 1; patients younger than 75 years of age with adequate oral intake and bone marrow, liver, cardiac and renal function; absence of other severe medical conditions or synchronous malignancy; and absence of contraindication for major surgery. Contraindications to inclusion were extra-abdominal disease, other malignancies and severe associated medical conditions made patients unfit for the protocol. Clinical or radiological progression after previous systemic chemotherapy was not considered an exclusion criterion. The presence of ovarian metastases was included because it is considered a manifestation of peritoneal disease [21]. Figure 1 shows the trial profile.

### Standardized data collection

The patients underwent a total body CT scan and gastric endoscopy with multiple biopsies to confirm primary GC. PM were diagnosed by systematic biopsies during laparoscopy. The presence of ascites was documented and analysed by wash cytology. Survival was calculated according to the Kaplan–Meier test. Quantitative variables are described as means.

### Surgical procedure and staging of PM

Laparoscopic exploration was performed with a 30° optic camera with the single incision laparoscopy surgery approach [22]. The extent of PM was assessed intraoperatively with Jacquet and Sugarbaker’s [23] PCI. The effects of bidirectional chemotherapy were evaluated by comparing the PCI before and after one cycle of bidirectional chemotherapy. Briefly, a single port was placed through the umbilicus. A senior surgeon who is an expert in PM evaluated the PCI of all patients. After evaluation of the peritoneal dissemination, quantification of ascites, and performance of cytology and peritoneal biopsies, a port system (Bard Port, C.R. Bard Inc., USA) was introduced into the abdominal cavity; the tip was placed on the *cul-de-sac* of Douglas, and the port was introduced through a 3-cm skin and fascia incision [24].

### Bidirectional chemotherapy

**Figure 2:**
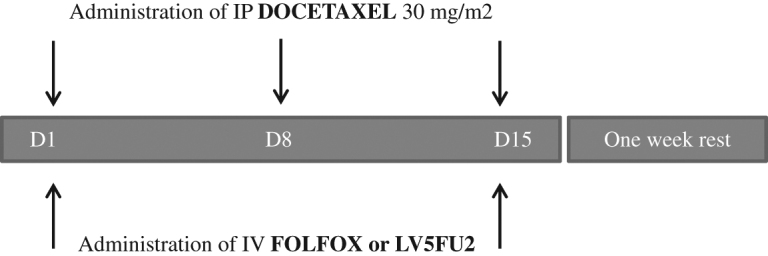
Schematic of one course of bidirectional systemic and intraperitoneal chemotherapy for peritoneal metastasis from gastric cancer. One course consisted of the following: docetaxel at 30 mg/m^2^ was administered intraperitoneally over 30 min in 1000 mL of saline on days 1, 8 and 15, IV folinic acid 200 mg/m^2^ was administered over 2 h, IV 5-fluoruracil (5-FU) was administered as a bolus of 400 mg/m^2^ and via continuous infusion at 600 mg/m^2^ on days 1 and 2, and IV oxaliplatin 85 mg/m^2^ was administered on days 1 and 15, followed by 7 days of rest. For patients with persistent neuropathy, FOLFOX was replaced by LV5FU2 (IV folinic acid administered at 200 mg/m^2^ in 250 mL of glucose perfusion on days 1 and 2, IV 5-fluoruracil (5-FU) administered as a bolus of 400 mg/m^2^ and via continuous infusion at 600 mg/m^2^ on days 1 and 2 without oxaliplatin). Before and after one course of bidirectional chemotherapy, 500 mL of saline solution was injected into the peritoneal cavity through the port, and fluid was recovered for cytology. After three courses corresponding to one complete cycle of bidirectional chemotherapy, the PCI response was evaluated with a second laparoscopy. IV, intravenous; IP, intraperitoneal; FOLFOX, folinic acid, fluorouracil and oxaliplatin; LV5FU2, folinic acid and fluorouracil.

All patients received one course of bidirectional chemotherapy in the Medical Oncological Department as follows: docetaxel 30 mg/m^2^ was administrated intraperitoneally over 30 min in 1,000 mL of saline on days 1, 8 and 15; IV folinic acid 200 mg/m^2^ was administered over 2 h; IV 5-fluoruracil (5-FU) was administered as a bolus of 400 mg/m^2^ and via a continuous infusion of 600 mg/m^2^ on days 1 and 2; and IV oxaliplatin 85 mg/m^2^ was administered on days 1 and 15 followed by 7 days of rest. For patients with persistent neuropathy, FOLFOX was replaced by LV5FU2 (IV folinic acid 200 mg/m^2^ was administered in 250 mL of glucose perfusion on days 1 and 2, IV 5-fluoruracil (5-FU) was administered as a bolus of 400 mg/m^2^ and via a continuous infusion at 600 mg/m^2^ on days 1 and 2 without oxaliplatin) (Figure 2). Before and after one course of bidirectional chemotherapy, 500 mL of saline solution was injected into the peritoneal cavity through the port, and fluid was recovered for cytology. Granulocyte colony-stimulating factor was administered at the investigator’s discretion. Good IP tolerance of bidirectional treatment was defined as the absence of abdominal pain, moderate IP tolerance was defined as the presence of abdominal pain that was controllable with mild analgesics, and poor IP tolerance was defined as requiring continuous IV perfusion of morphine. After three courses corresponding to one complete cycle of bidirectional chemotherapy, the PCI response was evaluated with a second laparoscopy. No cancer cells detected by biopsy after a cycle of bidirectional chemotherapy defined a complete response, a major response was defined as a decrease in PCI ≥ 25% of the initial value, and a minor response was defined as a decrease PCI less than 25% of the initial value. If a complete or major response was observed and the PM was evaluated as resectable, CRS and HIPEC were proposed. If a partial response or stability with unresectable PM was observed, treatment was repeated for three additional courses, followed by another laparoscopic evaluation. If progression was observed, it was proposed that the patient receive palliative care (i. e. a new line of systemic chemotherapy or the best supportive care).

### Endpoints

The primary endpoints were the feasibility and safety of combined bidirectional IV and IP chemotherapy. The secondary endpoints were OS, success of the combined therapeutic strategy, quality of life, complications related to laparoscopy and postoperative mortality. The Common Terminology Criteria for Adverse Events version 4.0 was applied to evaluate adverse drug reactions related to bidirectional chemotherapy [25]. OS was defined as the time from the diagnosis of PM to the time of death due to any cause. Success of the combined therapeutic strategy was defined as a 25% decrease in PCI between two laparoscopies after one cycle of bidirectional chemotherapy. A quality-of-life assessment was performed at patient registration and after the administration of each course of bidirectional chemotherapy treatment with QLQ C-29 and C30 [26, 27]. The complications of laparoscopy were defined according to the Dindo-Clavien classification [28]. All in-hospital complications were recorded. All patients were followed-up by clinical, biological and radiological evaluation until clinical progression and/or death, if it occurred.

## Results

### Patient characteristics

From March 2014 to March 2016, six consecutive patients were included in this study. Four of them were female (66.7%). The average age was 47 years [range 24–66]. Patient characteristics are summarized in Table 1.

**Table 1:** Demographic, clinical and histological characteristics of the patients included.

Patient	Sex	Age, years	Histology	Cytology	Her2+	PM Type	Prev-sCT	Bidirectional chemotherapy	
								IV	IP
1st	M	68	ADK	neg	neg	Recurrence	3+3 ECF, 12 FOLFOX	LV5FU2	DOC
2nd	F	48	ACDI, LP	+	neg	Synchronous	No	FOLFOX	DOC
3rd	M	42	ACDI, LP	+	1+	Synchronous	8 TEFOX	FOLFOX	DOC
4th	F	45	ACDI, LP	+	2+	Synchronous	No	FOLFOX	DOC
5th	F	24	ACDI, LP	+	2+	Synchronous	No	FOLFOX	DOC
6th	F	60	ACDI, LP	+	2+	Synchronous	8 TEFOX	LV5FU2	DOC

M, male; F, female; ADK, adenocarcinoma; ACDI, adenocarcinoma independent cells; LP, linitis plastica; Her2, human epidermal growth factor receptor 2; neg, negative; PM, peritoneal metastasis; Prev-sCT, previous systemic chemotherapy; IV, intravenous; IP, intraperitoneal; ECF, epirubicin, cisplatin and fluorouracil; FOLFOX, folinic acid, fluorouracil and oxaliplatin; TEFOX, docetaxel, 5-FU and oxaliplatin; LV5FU2, folinic acid and fluorouracil; DOC, docetaxel.

### Outcomes

PM was confirmed by histological biopsies in all patients: five of them had synchronous PM, and one had metachronous isolated PM after previous gastrectomy associated with perioperative systemic chemotherapy with platinum salts (cisplatin and oxaliplatin). Before bidirectional chemotherapy, the cytology of peritoneal fluid was positive in five patients (83%). Four patients had ascites at diagnosis (66%). All patients underwent one complete cycle of bidirectional chemotherapy; one patient had a second cycle. Four patients (66%) underwent a second laparoscopy; one patient had three laparoscopies. The tolerance of the IP treatment was good: abdominal pain during IP injection was described by two patients and controlled with mild analgesics. Four patients had good quality of life during bidirectional chemotherapy: one patient had an ECOG score of 0, and three patients had an ECOG score of 1 (Table 2). During the first cycle, two patients had grade 3–4 complications: one patient had grade 3 bone marrow suppression, and one patient had severe asthenia (Table 3). The adverse effects that occurred during the procedure are summarized in Table 4.

**Table 2:** Early and long-term outcomes for patients treated with bidirectional treatment.

Patient	Cycles of bidirectional chemotherapy	Tolerance of IP chemotherapy	ECOG status	1° Laparoscopy PCI (n=6)	2° Laparoscopy PCI (n=4)	Decrease ratio of PCI^a^	Results	CRS surgery	OS, months	Status
1st	1	Good	1	34	–	–	Progression	No	18	Alive
2nd	1.5	Moderate	2	30	12	60%	Major response^b^	No	5	Dead
3rd	1	Moderate	2	30	–	–	Progression	No	11	Dead
4th	1	Good	0	36	13	64%	Major response^b^	CRS+GT+HIPEC	16	Alive
5th	2	Good	1	39	29	26%	Major response^b^	No, Ovariectomy	14	Alive
6th	1	Good	1	32	18	44%	Major response^b^	No	15	Alive

IP, intraperitoneal; IP tolerance: good, absence of abdominal pain; moderate, presence of abdominal pain controlled with mild analgesics; ECOG, Eastern Cooperative Oncology Group; PCI, peritoneal cancer index; CRS, cytoreductive surgery; GT, total gastrectomy; HIPEC, hyperthermic intraperitoneal chemotherapy; OS, overall survival; Status, at the time of analysis (March 2016). ^a^ The decrease in the ratio of PCIs was calculated for each patient. ^b^Decrease≥25% of the initial peritoneal cancer index (PCI).

**Table 3:** Toxicities during bidirectional treatment.

Toxicity^a^					
Not IP catheter-related	Grade 1	Grade 2	Grade 3	Grade 4	Total
Anaemia	2	0	0	0	2
Leucopoenia	1	1	1	0	3
Febrile neutropenia	0	0	0	0	0
Thrombocytopenia	1	0	0	0	1
Asthenia	0	2	0	1	3
Diarrhoea	1	0	0	0	1
Neuropathy	0	1	0	0	1
Nausea/Vomiting	1	0	0	0	1
Renal	0	0	0	0	0
Metabolic	0	0	0	0	0
Total	6	4	1	1	12

IP, intraperitoneal. ^a^ Toxicity was assessed during bidirectional treatment according to the National Cancer Institute (NCI-CTC).

**Table 4:** Adverse effects during bidirectional treatment.

Adverse effects				
Catheter-related	1st cycle (n=6)	2nd cycle (n=4)	3rd cycle (n=1)	Total
IP catheter infection	1	0	0	1
IP catheter blocked	0	0	0	0
Access problems	0	0	0	0
**Possibly IP treatment-related**				
Other infection	0	0	0	0
Abdominal pain	1	1	1	3
Patient refusal	0	0	0	0
Bowel complication/peritonitis	0	0	0	0
Refractory ascites	4	2	1	7
Paracentesis	4	1	1	6
Severe malnutrition	2	0	1	3
Total	12	4	4	20

IV, intravenous; IP, intraperitoneal; PCI, peritoneal cancer index; cycle, bidirectional chemotherapy corresponding to three consecutive courses of docetaxel 30 mg/m^2^ administered intraperitoneally over 30 min in 1000 mL of saline on days 1, 8 and 15, IV folinic acid 200 mg/m^2^ administered over 2 h, IV folinic acid 200 mg/m^2^ administered over 2 h, IV 5-fluoruracil (5-FU) administered as a bolus of 400 mg/m^2^ on day 1 and via a continuous infusion at 600 mg/m^2^ on days 1 and 2, and IV oxaliplatin 85 mg/m^2^ administered on days 1 and 15, followed by 7 days of rest.

**Figure 3:**
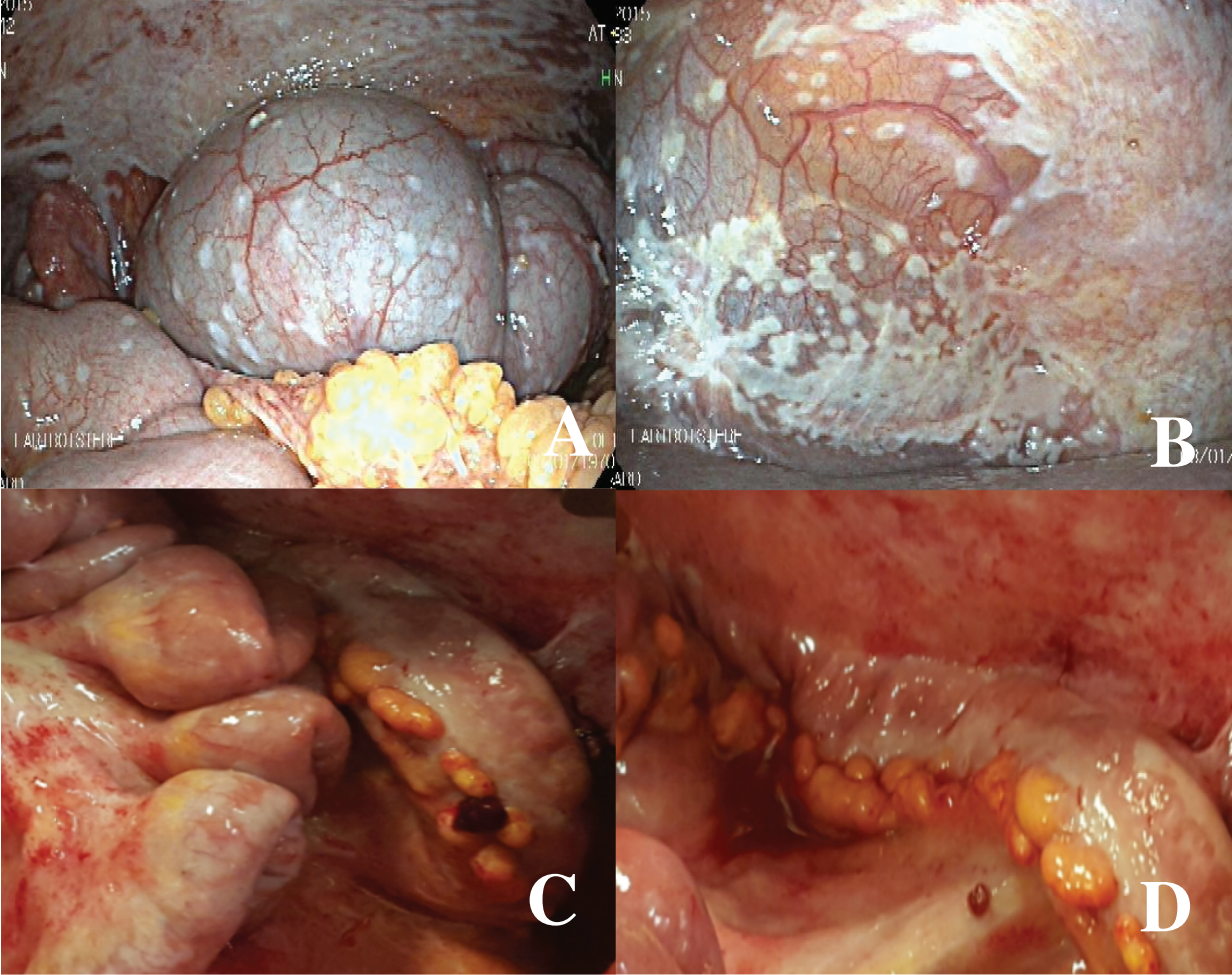
Laparoscopy before and after bidirectional treatment. The first laparoscopy (upper) for staging shows the peritoneal metastases in the right subphrenic peritoneum (left, A) and the pelvis (right, B). The second laparoscopy (lower), after bidirectional treatment, shows the major response of peritoneal metastases in the small bowel (left, C) and in the left parietal peritoneum (right, D). Directed biopsies in the parietal peritoneum (D) showed a major histological response (peritoneal regression grading score (PRGS) 2, major regression features, few residual tumour cells). Figures C and D show chemical peritonitis due to the effects of direct contact with intraperitoneal chemotherapy during laparoscopy.

The median follow-up was 13 months, the median OS was 13 months [range 5–18 months], and the 1-year OS was 67%. Four patients were alive at the time of the analysis (March 2016). The decrease in PCI between the first and second laparoscopy after one cycle of bidirectional chemotherapy is shown in Table 2. After the first bidirectional cycle, one patient had a major histological response (peritoneal regression grading score 2, major regression features, few residual tumour cells) detected by eight biopsies from four abdominal regions; the PCI decreased by 64% of the initial value, and the patient underwent CRS with HIPEC with a curative intent (Figure 3). Two patients (2nd and 6th) had major macroscopic responses with PCIs of 60% and 44% of their initial values, respectively. However, the PM remained unresectable, and they died from chronic occlusive symptoms and severe malnutrition. One patient had a major response with a PCI of 26% of its initial value. However, at the second laparoscopy, the PM was judged unresectable; consequently, she underwent a second cycle of bidirectional chemotherapy with progression of PM diagnosed by the third laparoscopy. Two patients had progression of disease. After one bidirectional cycle, peritoneal biopsies were positive in all patients and reverted to a negative histology in three patients. Peritoneal cytology became negative in three of five patients with previous positive cytology. The volume of ascites decreased in one patient.

## Discussion

PM from GC was considered a terminal event [29]. Patients who are not amenable to curative resection generally receive palliative chemotherapy to control related symptoms and improve OS [1]. Despite new drug regimens, emerging strategy data and improved understanding of tumour biology, OS remains poor in metastatic GC [30, 31, 32]. A multimodal approach including neoadjuvant systemic chemotherapy followed by surgery appears to be a reasonable strategy for tumour downstaging and sterilization of micrometastases to improve OS. Two randomized trials comparing perioperative chemotherapy with surgery alone showed the efficacy of this approach in resectable GC [33, 34]. More recently, the FLOT regimen has emerged as a new therapeutic standard in the perioperative setting [4, 5]. Unfortunately, systemic neoadjuvant chemotherapy has never significantly downstaged peritoneal seeding, and many consider it an inadequate therapeutic option for PM [1]. Recent studies have suggested that radical resection of macroscopic disease and perioperative chemotherapy to treat microscopic disease could be a potentially curative treatment for advanced GC with limited PM [35, 36]. Locoregional therapeutic approaches combining CRS with perioperative intraperitoneal chemotherapy suggest improved survival [8]. Glehen et al. [36] showed that the median OS was 9.2 months, and the 1-, 3- and 5-year survival rates were 43, 18 and 13%, respectively. Moreover, there is still no therapeutic standard for IP treatment for gastric PM. Accordingly, because it is currently done unresectable colorectal liver metastases [37, 38], neoadjuvant IP chemotherapy has been proposed as a treatment modality to increase the rate of patients with peritoneal seeding from GC who obtain complete clearing of the peritoneal dissemination [9]. Neoadjuvant IV chemotherapy combined with IP chemotherapy without hyperthermia has shown its efficacy with an acceptable toxicity profile in Japanese trials [12, 13, 14, 15, 16, 17, 18]. However, in Caucasian patients, the efficacy of this bidirectional treatment remains to be evaluated. To our knowledge, this is the first Western study to evaluate the safety and efficacy of combined IP and IV chemotherapy for non-resectable PM from GC. Sgarbura et al. [39] tested IP oxaliplatin in the neoadjuvant setting in patients presenting with unresectable PC of colorectal origin with encouraging results. The effects of IP taxane administration were analysed in phase II and III studies in advanced GC [12, 13, 16, 17]. Pharmacokinetic studies have shown that IP chemotherapy provides high concentrations of a cytotoxic agent directly to the peritoneal space [35, 40, 41] with low systemic effects. However, effective concentrations of systemic drugs are achievable via absorption of the agent through the lymphatic stoma located on the peritoneal surface [42]. Taxanes are hydrophobic and high molecular weight drugs that remain at a high IP concentration for 48–72 h in contact with the peritoneal nodules, producing anti-tumour effects and making them ideal chemotherapeutic agents for IP administration (Table 5). Morgan et al. [43] established that administration of IP docetaxel could be safely delivered at a dose of 100 mg/m^2^ every 3 weeks. According to phase I studies, the recommended doses of IP docetaxel combined with oral cancer drugs (TS-1) are 45–60 mg/m^2^ [17, 44]. Yonemura et al. [11], using dual IP anticancer drugs, lowered the concentration of IP docetaxel to 30 mg/m^2^ with mild toxicity. Similarly, we used a concentration of 30 mg/m^2^ of docetaxel to reduce toxicity when administered in association with IV FOLFOX. According to previous reports [13, 45] and in our study, the haematological and non-haematological toxicities correlated with systemic chemotherapy and adverse effects after bidirectional chemotherapy were acceptable. No chemotherapy-related deaths were experienced. During the laparoscopies, no abdominal adverse effects were reported, except for one intraperitoneal port infection resolved by conservative treatment. Abdominal pain was controlled with mild analgesics. However, repeated paracentesis for refractory ascites was necessary for four patients, with consequent severe malnutrition for one of them. Notably, inflammation of the peritoneal serosa, observed after IP chemotherapy and confirmed by histology, seems to be the cause of this refractory ascites.

**Table 5:** Pharmacokinetic parameters for docetaxel.

Docetaxel 40 mg	
Molecular weight (daltons)	861.9
AUC peritoneal/plasma ratio	207^a^–552
Drug penetration distance with IP administration	NA
Recommended IV dose (mg/m^2^)	100
Recommended IP dose (mg/m^2^)^b^	45–60

AUC, area under curve; IV, intravenous; IP, intraperitoneal; mg, milligrams.

^a^ in hyperthermic chemoperfusion; ^b^ combined with oral cancer drugs.

Fava et al. [46] claimed that the most important effect of bidirectional chemotherapy based on taxanes seems to be the high response rate in PM extent. In our experience, at the first laparoscopy, the mean PCI was 34 [range 30–39], and it decreased to 18 [range 12–29] after the first bidirectional cycle, which was 48% less than the initial PCI (Table 2). This surprising result was better than the planned cut-off of 25% and suggested possible clinical benefits of bidirectional chemotherapy for PM of GC. We observed a high negative conversion rate of peritoneal cytology and histology (60% and 50%, respectively). Furthermore, the median OS was 13 months [range 5–18]; the 1-year OS rate was 67%. After a major response, one patient underwent CC0 cytoreduction followed by HIPEC with oxaliplatine for 30 min. In the study of Yonemura et al. [10], 30 of 61 enrolled patients underwent surgery, and 14 of them were disease-free with long-term survival (20.4 and 15 months of OS, respectively) and without major toxicities. However, in our study, we enrolled patients with high volume of carcinomatosis (PCI>15) and initial unresectable PM, while the heterogeneity of the population of Asiatic patients (i. e. patients with limited macroscopic PM and patients with only positive cytology without macroscopic PM) was probably responsible for the better results of Yonemura’s study in terms of OS. In the early study, bidirectional chemotherapy was able to eradicate free cancer cells in the peritoneal cavity.

In this study, we evaluated the macroscopic response to bidirectional chemotherapy by laparoscopy. Preoperative radiologic evaluation is considered inaccurate to assess the PCI and resectability of PM, and laparoscopy is mandatory [22, 47] and used to place a peritoneal access chamber. If IP chemotherapy is performed during the perioperative period when adhesions have not yet developed, the entire abdominal cavity can be treated equally. The number of bidirectional chemotherapy cycles depends on the effect on tumours, and an accurate preoperative evaluation of PM is mandatory to propose secondary curative CRS.

In phase II trials, Ishigami et al. [14] showed 1-year OS rates of 77% and 78% of patients with PM from GC treated weekly with bidirectional IP and IV paclitaxel combined with S-1. These promising results have not been confirmed in the PHOENIX-CG phase III trial despite a prolongation of the MST by 2.5 months and a negative conversion rate (78%) in peritoneal cytology [12]. Although bias was present due to the baseline imbalance between the arms regarding the extent of PM, amount of ascites and crossover, in the exploratory analyses, the Japanese study suggested possible clinical benefits of bidirectional chemotherapy.

Though it has been relegated to being a palliative procedure, the novel drug delivery system PIPAC (pressurized intraperitoneal aerosol chemotherapy) was analysed in a recent review by Garg et al. [48]. Among 79 papers included, only one study [49] highlighted the effects of repeated PIPAC in the neoadjuvant setting to downgrade the PCI. However, in this retrospective cohort of 21 patients treated with secondary CRS and HIPEC after PIPAC, there were only three patients with PM from GC associated with the worst prognosis. Future clinical trials evaluating the potential place of PIPAC as a neoadjuvant therapy in advanced GC with synchronous peritoneal recurrences are expected.

## Conclusions

A combination of IV and IP chemotherapy should be considered in patients with PM from gastric cancer. Accordingly, bidirectional chemotherapy appears to be safe and could be proposed in the pre-operative setting in highly selected patients. Bidirectional chemotherapy should be evaluated more extensively in phase I–II studies.
